# Supplementary material for: RNA switch model for localization and translation of the myelin basic protein mRNA
Source: bioRxiv. 2025 Nov 20:2025.11.19.689361. Preprint. [Version 1] doi: 10.1101/2025.11.19.689361 (PMC12667860; doi:10.1101/2025.11.19.689361)
Supplement: Supplement 5 [file media-5.pdf]

Supporting Information

Table S1. Significantly enriched and de-enriched proteins in MLS pulldown study

Gene name	Protein name	log_2FoldChange	p_{adj}
Tars3	Threonine-tRNA ligase	1.59915872	0.00160976
Hnrnpf	Heterogeneous nuclear ribonucleoprotein F	1.47606397	0.00169003
Cct4	T-complex protein 1 subunit delta	1.11260304	0.00169003
Cct5	T-complex protein 1 subunit epsilon	1.10858645	0.00044553
Cct2	T-complex protein 1 subunit beta	1.09223648	0.00080849
Cct7	T-complex protein 1 subunit eta	1.07034113	0.00169003
Cct8	T-complex protein 1 subunit theta	0.9961152	0.00106049
Cct6a	Chaperonin containing TCP1 subunit 6A	0.94039895	0.0065329
Aimp1	Aminoacyl tRNA synthetase complex-interacting multifunctional protein 1	0.92121638	0.02619575
Lars1	Leucine-tRNA ligase	0.85600888	0.007258
Cct3	T-complex protein 1 subunit gamma	0.8403121	0.01653504
Tcp1	T-complex protein 1 subunit alpha	0.68643992	0.06476284
Hadhb	Trifunctional enzyme subunit beta, mitochondrial	−1.1105623	0.00478055
Igf2bp2	Insulin-like growth factor 2 mRNA binding protein 2	−1.4783708	0.009356
Abcf1	ATP-binding cassette sub-family F member 1	−1.5946004	0.00044553
Snrnp70	U1 small nuclear ribonucleoprotein 70 kDa	−2.0235793	0.00809546
Msi2	Musashi RNA-binding protein 2	−2.165768	0.00654469
Sf3b2	Splicing factor 3b, subunit 2	−2.1681579	0.00545222
Sf3b3	Splicing factor 3b, subunit 3	−2.1719484	0.00106049

Dataset S01 (XLSX) Supporting Information Dataset S01_SLAP-seq
Dataset S02 (CSV) Supporting Information Dataset S02_Proteomics Raw Data
Dataset S03 (XLSX) Supporting Information Dataset S03_Species Sequence Alignment
Dataset S04 (XLXS) Supporting Information Dataset S04_Synthesized Oligonucleotides
